# CYP1B1 Polymorphisms and Susceptibility to Prostate Cancer: A Meta-Analysis

**DOI:** 10.1371/journal.pone.0068634

**Published:** 2013-07-04

**Authors:** Hongtuan Zhang, Liang Li, Yong Xu

**Affiliations:** 1 Department of Urology, Second Affiliated Hospital of Tianjin Medical University, Tianjin Key Institute of Urology, Tianjin, China; 2 Laboratory of Population and Quantitative Genetics, School of Life Sciences, Tianjin Medical University, Tianjin, China; The University of Texas MD Anderson Cancer Center, United States of America

## Abstract

**Background:**

Studies investigating the association between single-nucleotide polymorphisms (SNPs) of the cytochrome P450 1B1 (CYP1B1) and prostate cancer (PCa) risk report conflicting results. To derive a more precise estimation of the relationship between CYP1B1 polymorphisms and PCa risk, a meta-analysis was performed.

**Methodology/Principal Findings:**

A comprehensive literature search was conducted to identify all eligible studies of CYP1B1 polymorphisms and PCa risk. A total of 14 independent studies, including 6380 cases and 5807 controls, were identified. We investigated by meta-analysis the effects of 5 polymorphisms in CYP1B1 L432V (12 studies, 5999 cases, 5438 controls), R48G (6 studies, 1647 cases, 1846 controls), N453S (4 studies, 1407 cases, 1499 controls), −13C/T (4 studies, 1116 cases, 1114 controls), and A119S (4 studies, 1057 cases, 1018 controls). There was no evidence that L432V had significant association with PCa in overall population. After subgroup analyses by ethnicity, we found that L432V was significantly associated with PCa risk in Asians (additive: OR = 2.38, 95%CI = 1.31-4.33, P = 0.004; recessive: OR = 2.11, 95%CI = 1.17–3.79, P = 0.01; dominant: OR = 1.52, 95%CI = 1.14–2.01, P = 0.004; allelic: OR = 1.52, 95%CI = 1.20–1.92, P = 0.0006). When stratified by source of controls, significantly elevated PCa risk was found in all genetic models in population based studies (additive: OR = 1.34, 95%CI = 1.14–1.57, P = 0.0003; recessive: OR = 1.25, 95%CI = 1.09–1.43, P = 0.002; dominant: OR = 1.25, 95%CI = 1.11–1.41, P = 0.0002; allelic: OR = 1.18, 95%CI = 1.09–1.28, P<0.0001). For N453S, there was a significant association between N453S polymorphism and PCa risk in both overall population (dominant: OR = 1.18, 95%CI = 1.00–1.38, P = 0.04) and mixed population (domiant: OR = 1.31, 95%CI = 1.06–1.63, P = 0.01; allelic: OR = 1.27, 95%CI = 1.05–1.54, P = 0.01). For A119S, our analysis suggested that A119S was associated with PCa risk under recessive model in overall population (OR = 1.37, 95%CI = 1.04–1.80, P = 0.03).

**Conclusions:**

The results suggest that L432V, N453S, and A119S polymorphisms of CYP1B1 might be associated with the susceptibility of PCa. Further larger and well-designed multicenter studies are warranted to validate these findings.

## Introduction

Prostate cancer (PCa) is one of the most frequently diagnosed malignancies and a common cause of cancer mortality in men in the Western hemisphere [1], [2], which has become a major public health challenge. The mechanism of its carcinogenesis, like other cancers, is still not fully understood. Identifying risk factors for PCa is critically important to develop potential interventions and to expand our understanding of the biology of this disease. As with other complex diseases, PCa is caused by both genetic and environmental factors. Genetic factors, including the sequence alterations and organization aberrations of the cellular genome that range from single-nucleotide substitutions to gross chromosome, could modulate several important biological progress and alert susceptibility to PCa consequently. Single nucleotide polymorphisms (SNPs) have attracted considerable attention in recent years as potential markers for predicting disease susceptibility and for guiding individualized therapeutic regimens. It is well known that steroid hormones play a fundamental role in the pathogenesis of PCa. The prostate is an androgen-dependent organ and polymorphic variants in a number of genes involved in androgen metabolism have been implicated in PCa risk. For example, several studies results showed that steroid 5-alpha-reductase 2, cytochrome P450 3A4 (CYP3A4), and CYP3A5 variants may influence risk of developing PCa or more aggressive disease [3], [4]. In overall, recent studies suggest that genetic polymorphisms of genes involved in estrogen bioactivation and detoxification, including CYP1B1, might impact susceptibility to PCa.

The CYP1B1 gene is located on chromosome 2p21–22 [5], [6]. The gene contains three exons (371, 1044 and 3707 bp) and two introns (390 and 3032 bp) [5], [7], [8]. CYP1B1 is transcriptionally induced by compounds such as 2,3,7,8-tetrachlorodibenzo-p-dioxin or dioxin, and regulated by several key transcriptional factors including oestrogen receptor and aryl hydrocarbon receptor [5]. Apart from its role in xenobiotic metabolism, CYP1B1 is implicated in the bioactivation of pro-carcinogens [7], [9], [10]. The enzyme also appears to play a role in the metabolism of certain anticancer agents used in the treatment of hormone-induced cancers [11]. The CYP1B1 is involved in the activation of many procarcinogens and the hydroxylation of testosterone, and therefore variations in CYP1B1 may lead to higher susceptibility to PCa. In the past years, L432V (Leu432Val, rs1056836, 4326C/G), R48G (Arg48Gly, rs10012, 142C/G), N453S (Asn453Ser, rs1800440, 4390A/G), −13C/T (RS2617266), and A119S (Ala119Ser, RS1056827, 355G/T) polymorphisms have attracted widespread attention. Of the most studied SNPs, four are reported to result in amino acid substitutions, and they are L432V, R48G, N453S, and A119S. Importantly, these polymorphic variants have been associated with enhanced catalytic activity when compared to the wild-type allele [12], [13], it has been postulated that this functional finding may confer susceptibility toward cancer at a certain extent [13].

An increasing number of case-control studies were performed to identify the association of these polymorphisms with PCa risk. However, these studies have appeared in the literature either supporting or negating the significant association. A single study might not be powered sufficiently to detect a small effect of the polymorphisms on PCa, particularly in relatively small sample sizes. Various types of study populations and study designs might also have contributed to these disparate findings. 2 previously published meta-analyses regarding the association of CYP1B1 L432V with PCa susceptibility were performed to provide evidence for or against an association of this polymorphism with cancer risk [14]–[15]. However, the 2 previous meta-analyses did not study other 4 polymorphisms. Hence, we performed a meta-analysis of 5 common polymorphisms in CYP1B1 involved in the androgen and estrogen metabolic pathways to determine their potential associations with PCa risk.

## Materials and Methods

### Publication Search

PubMed, Cochrane Library, and Embase electronic databases were searched using the search terms: “CYP1B1” or “cytochrome P450 1B1”, “polymorphism” or “variation”, and “PCa” (last search was updated on 9 February 2013). We evaluated all the retrieved publications to retain the most eligible studies. Additional studies were selected by searching related reference articles for data involving the association between the CYP1B1 polymorphisms with PCa risk in a case-control design. Only published studies with full text articles were included. Authors were contacted directly regarding crucial data not reported in original articles. When overlapping data of the same patient population were included in more than one publication, only the most recent or complete study was used in this meta-analysis.

### Inclusion and Exclusion Criteria

The following inclusion criteria were used to select literatures for the meta-analysis: (1) about CYP1B1 polymorphisms and PCa risk; (2) case-control studies; and (3) sufficient genotype data were presented to calculate odds ratio (OR) with 95% confidence interval (CI) (the article provided the sample size, distribution of alleles, genotypes or other information that could help to infer study characteristics). Major reasons for exclusion of studies were: (1) no control population; (2) reviews and duplication of the previous publication; (3) no usable data reported; (4) not involving the CYP1B1 gene; and (5) animal studies.

### Data Extraction

From the eligible literature, two authors independently selected data according to the inclusion criteria outlined above. Any disagreement was resolved by discussion between the two authors. If they could not reach a consensus, another author participated in the discussion and a final decision was made by the majority. The following data were extracted: the last name of the first author, publication year, country in which study was conducted, ethnicity of the population, source of controls (population- or hospital-based), Hardy-Weinberg equilibrium (HWE), available genotype, and number of PCa cases and controls studied. Ethnic group was defined as Asian, African, Caucasian or “mixed”, including more than one ethnic category.

### Meta-analysis

All statistical tests performed in this study were two-tailed and p values less than 0.05 were considered significant, unless otherwise stated. Statistical analyses were performed with Review Manage, version 5.0 and Stata 10.0. We assessed the departure from the HWE for the control group in each study using an online HWE calculator (http://ihg.gsf.de/cgi-bin/hw/hwa1.pl).

Crude ORs with 95% CIs were calculated to assess the strength of the association between CYP1B1 L432V polymorphism and PCa risk. We explored the association for additive model (GG VS CC), recessive model (GG vs GC+CC), dominant model (GG+GC vs CC) and allelic contrast (C vs G). For other polymorphisms, we evaluated the same effects. Subgroup analyses were also performed by ethnicities and source of controls. Bonferroni correction for multiple testing was also applied.

The evaluation of the meta-analysis results included an examination of the heterogeneity, an analysis of the sensitivity, and an examination for bias. Heterogeneity assumption was checked by the chi-square-based Q-test. A P value of more than 0.10 for the Q-test indicates a lack of heterogeneity among the studies. Either a random-effects model or fixed-effects model was used to calculate pooled effect estimates in the presence or absence of heterogeneity [16], [17]. The one-way sensitivity analyses were performed to assess the stability of the results, namely, a single study in the meta-analysis was deleted each time to reflect the influence of the individual data set to the pooled OR. An estimate of potential publication bias was carried out by the funnel plot and the Egger’s linear regression test. An asymmetric funnel plot suggests a possible publication bias. Then, the funnel plot asymmetry was assessed by the Egger’s linear regression test, and the significance of the intercept was determined by the t-test suggested by Egger.

## Results

### Eligible Studies

**Figure 1:**
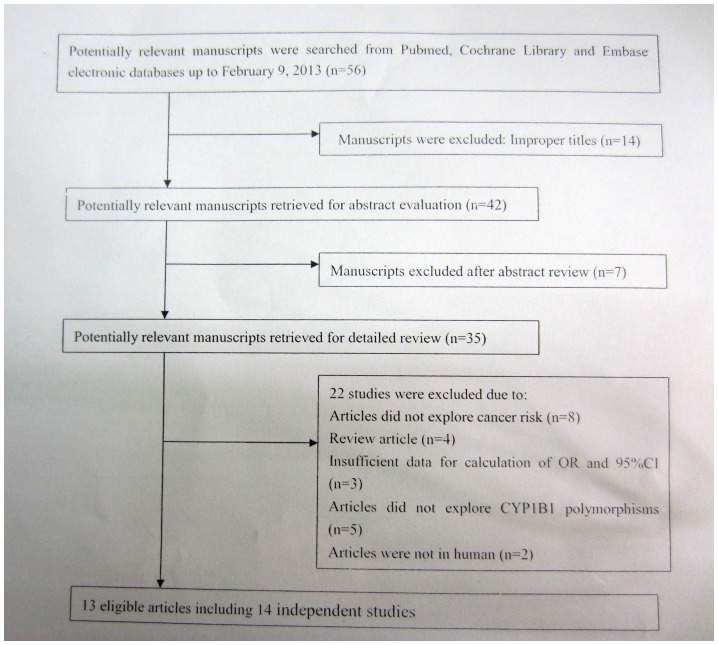
The flow diagram for the review process and outcomes of inclusion and exclusion.

The process of selection of studies for inclusion in the meta-analysis is summarized in Figure 1. The database search identified 56 potentially relevant citations, of which 42 were judged to be of potential interest on the basis of the title. On the basis of the abstract, 35 studies were reviewed in their entirety. During the extraction of data, 22 articles were excluded (2 were not in human; 3 did not provide sufficient data for calculation of OR and 95%CI; 8 did not explore PCa risk; 5 did not explore CYP1B1 gene polymorphisms; 4 were reviews), leaving 13 articles identified with criteria for inclusion and exclusion [18]–[30]. 1 article examined the association in independent populations and thus was treated as two separate studies [22]. Finally, we identified 14 independent studies in 13 eligible reports [18]–[30], including 6380 cases and 5807 controls. There were 6 groups of Caucasians [18], [20], [22], [23], [30], 3 of Asians [25], [27], [29], 1 of Africans [21], and 4 of mixed population [19], [24], [26], [28]. 5 studies were population-based [18], [19], [25], [29], [30] and 9 studies were hospital-based [20]–[24], [26]–[28]. All the included 14 studies were written in English. Main characteristics for all eligible studies were listed in tables 1 and 2.

**Table 1:** Main characteristics of studies included in this meta-analysis.

First author	Year	Country	Cases	Controls	Ethnicity	Polymorphisms
Tang et al.	2000	USA	50	50	Caucasian	L432V
Tanaka et al.	2002	Japan	117	200	Asian	−13C/T, R48G, A119S, L432V, N453S
Chang et al.	2003	USA	404	222	Mixed	−13C/T, R48G, A119S, L432V, N453S
Fukatsu et al.	2004	Japan	136	255	Asian	L432V
Cicek et al.	2005	USA	439	479	Mixed	A119S, L432V
Sobti et al.	2006	India	100	100	Asian	L432V
Cussenot et al.	2007	French	1053	837	Caucasian	L432V
Berndt et al.	2007	USA	488	617	Mixed	R48G, L432V, N453S
Beuten et al.	2008	USA	153	240	Hispanic Caucasian	−13C/T. R48G, L432V, N453S
Beuten et al.	2008	USA	496	498	Non-Hispanic Caucasian	−13C/T. R48G, L432V, N453S
Beuten et al.	2009	USA	67	133	African American	R48G
Rodrigues et al.	2011	Brazil	154	154	Caucasian	A119S
Catsburg et al.	2012	USA	1419	756	Mixed	L432V
Holt et al.	2013	USA	1304	1266	Caucasian	L432V

**Table 2:** Distribution of CYP1B1 genotype among prostate cancer cases and controls included in the meta-analysis.

SNP	First author	Cases			HWE	Controls			Control source
		AA	Aa	aa		AA	Aa	aa	
L432V	Tang et al.	33		17		44		6	PB
Leu432Val	Tanaka et al.	73	35	9	No	141	46	13	PB
rs1056836	Chang et al.	94	169	47	Yes	53	98	31	HB
4326C/G	Fukatsu et al.	87	42	7	Yes	180	72	3	HB
	Cicek et al.	145	185	109	No	151	205	123	HB
	Sobti et al.	53	34	13	Yes	69	26	5	PB
	Cussenot et al.	352	511	190	Yes	315	395	127	HB
	Berndt et al.	159	235	92	Yes	166	313	132	HB
	Beuten et al.- Hispanic	83	55	4	Yes	130	85	22	HB
	Beuten et al.- Non-Hispanic	164	231	96	Yes	161	234	101	HB
	Catsburg et al.	398	619	402	No	231	335	190	PB
	Holt et al.	375	624	257	Yes	435	585	215	PB
R48G	Tanaka et al.	53	49	15	No	99	72	29	PB
rs10012	Chang et al.	190	124	31	Yes	86	82	16	HB
142C/G	Berndt et al.	213	194	62	No	262	247	84	HB
	Beuten et al.- Hispanic	76	66	11	Yes	113	105	22	HB
	Beuten et al.- Non-Hispanic	262	189	45	No	275	175	46	HB
	Beuten et al.-2009	13	39	15	Yes	43	58	32	HB
N453S	Tanaka et al.	117	0	0	Yes	200	0	0	PB
rs1800440	Chang et al.	203	101	10	Yes	132	46	4	HB
4390A/G	Berndt et al.	324	145	15	Yes	437	157	14	HB
	Beuten et al.- Hispanic	104	41	4	Yes	170	57	3	HB
	Beuten et al.- Non-Hispanic	299	142	19	Yes	309	148	22	HB
−13C/T	Tanaka et al.	58	50	9	Yes	97	86	17	PB
rs2617266	Chang et al.	195	126	29	Yes	87	80	16	HB
	Beuten et al.- Hispanic	80	63	10	Yes	123	95	21	HB
	Beuten et al.- Non-Hispanic	265	188	43	No	270	176	46	HB
A119S	Tanaka et al.	58	42	17	No	151	38	11	PB
355G/T	Chang et al.	186	131	32	Yes	86	83	16	HB
rs1056827	Cicek et al.	216	178	43	Yes	247	197	35	HB
	Rodrigues et al.	40	60	54	No	54	52	48	HB

CYP1B1, cytochrome P450 1B1; SNP, single nucleotide polymorphism; HWE, Hardy–Weinberg equilibrium; “A” denotes a common allele; “a” denotes a rare allele; PB, population based; HB, hospital based.

### Meta-Analysis

The detailed results of this meta-analysis and heterogeneity test were presented in Table 3. The positive results remained significant after adjusted by multiple testing. When the Q-test of heterogeneity was not significant, we conducted analyses using the fixed effect models. The random effect models were conducted when we detected significant between-study heterogeneity.

**Table 3:** Meta-Analysis of CYP1B1 Polymorphisms and Prostate Cancer.

Study	No	Sample size		Additive model			Recessive model			Dominant model			Allelic contrast		
		Case	Control	OR (95% CI)	P	Ph	OR (95% CI)	P	Ph	OR (95% CI)	P	Ph	OR (95% CI)	P	Ph
L432V															
Total	12	5999	5438	1.10 (0.88–1.37)	0.40	0.001	1.11 (0.92–1.32)	0.27	0.006	1.09 (0.96–1.24)	0.19	0.02	1.07 (0.96–1.19)	0.22	0.001
**Ethnicities**															
Mixed	4	2654	2028	0.95 (0.73–1.23)	0.67	0.08	1.02 (0.89–1.17)	0.75	0.26	0.97 (0.85–1.10)	0.62	0.13	0.97 (0.85–1.12)	0.68	0.06
Caucasian	5	2992	2855	1.09 (0.78–1.53)	0.61	0.01	1.13 (0.83–1.54)	0.45	0.009	1.16 (1.04–1.29)	0.008	0.15	1.06 (0.92–1.22)	0.41	0.03
Asian	3	353	555	2.38 (1.31–4.33)	0.004	0.22	2.11 (1.17–3.79)	0.01	0.21	1.52 (1.14–2.01)	0.004	0.58	1.52 (1.20–1.92)	0.0006	0.45
**Source of controls**															
HB	7	3057	3097	0.94 (0.70–1.28)	0.71	0.005	0.97 (0.76–1.22)	0.78	0.03	1.00 (0.90–1.11)	0.99	0.11	0.98 (0.87–1.11)	0.79	0.02
PB	5	2942	2341	1.34 (1.14–1.57)	0.0003	0.34	1.25 (1.09–1.43)	0.002	0.13	1.25 (1.11–1.41)	0.0002	0.28	1.18 (1.09–1.28)	<0.0001	0.14
R48G															
Total	6	1647	1846	0.96 (0.76–1.20)	0.71	0.87	0.93 (0.75–1.15)	0.48	0.99	1.00 (0.87–1.14)	0.98	0.13	0.98 (0.89–1.09)	0.72	0.53
**Ethnicities**															
Mixed	2	814	777	0.90 (0.65–1.25)	0.53	0.93	0.95 (0.70–1.29)	0.74	0.75	0.87 (0.71–1.06)	0.17	0.20	0.91 (0.78–1.06)	0.24	0.38
Caucasian	2	649	736	0.95 (0.64–1.39)	0.78	0.48	0.92 (0.63–1.33)	0.66	0.59	1.05 (0.85–1.30)	0.66	0.39	1.01 (0.86–1.20)	0.88	0.37
**Sorce of controls**															
HB	5	1530	1646	0.96 (0.75–1.21)	0.71	0.77	0.93 (0.75–1.17)	0.55	0.98	0.98 (0.79–1.22)	0.81	0.09	0.97 (0.87–1.09)	0.63	0.42
N453S															
Total	4	1407	1499	1.22 (0.80–1.86)	0.36	0.59	1.17 (0.77–1.79)	0.46	0.67	1.18 (1.00–1.38)	0.04	0.37	0.97 (0.56–1.68)	0.92	<0.00001
Mixed	2	798	790	1.50 (0.80–2.80)	0.21	0.87	1.39 (0.74–2.59)	0.30	0.91	1.31 (1.06–1.63)	0.01	0.58	1.27 (1.05–1.54)	0.01	0.61
Caucasian	2	609	709	1.02 (0.57–1.82)	0.95	0.29	1.01 (0.57–1.80)	0.96	0.31	1.04 (0.82–1.31)	0.76	0.40	0.73 (0.32–1.63)	0.44	0.0005
−13C/T															
Total	4	1116	1114	0.87 (0.64–1.20)	0.40	0.94	0.89 (0.66–1.20)	0.45	0.96	0.94 (0.80–1.12)	0.50	0.39	0.94 (0.82–1.08)	0.39	0.62
**Ethnicities**															
Caucasian	2	649	731	0.89 (0.60–1.32)	0.57	0.58	0.87 (0.59–1.27)	0.47	0.60	1.03 (0.84–1.28)	0.76	0.71	0.99 (0.84–1.17)	0.93	0.62
**Source of controls**															
HB	3	999	914	0.87 (0.62–1.22)	0.42	0.83	0.89 (0.64–1.23)	0.47	0.85	0.94 (0.78–1.13)	0.51	0.22	0.94 (0.81–1.09)	0.40	0.41
A119S															
Total	4	1057	1018	1.59 (0.97–2.61)	0.07	0.05	1.37 (1.04–1.80)	0.03	0.22	1.38 (0.81–2.34)	0.23	<0.0001	1.33 (0.89–1.98)	0.16	<0.0001
**Ethnicities**															
Mixed	2	786	664	1.21 (0.82–1.79)	0.33	0.31	1.26 (0.87–1.84)	0.23	0.51	0.96 (0.78–1.19)	0.72	0.11	1.02 (0.87–1.20)	0.80	0.13
**Source of controls**															
HB	3	940	818	1.30 (0.95–1.80)	0.10	0.49	1.23 (0.92–1.66)	0.16	0.79	1.05 (0.75–1.49)	0.76	0.06	1.07 (0.93–1.24)	0.33	0.13

CYP1B1, cytochrome P450 1B1; OR, odds ratio; CI, confidence interval. Ph, P value for heterogeneity; PB, population based; HB, hospital based;

#### L432V

12 independent studies with a total of 5999 cases and 5438 controls were included in the meta-analysis for L432V polymorphism. The Q-test of heterogeneity was significant and we conducted analyses using random effect models in overall population. After subgroup analyses by ethnicity, significant heterogeneity was effectively removed in Asians and mixed population. In subgroup analyses stratified by source of controls, the Q-test of heterogeneity was significant and we conducted analyses using random effect models except in the dominant model in hospital based studies. In overall population analyses, there was no significant association between L432V polymorphism and PCa susceptibility when examining additive, recessive, dominant, and allelic contrasts. In subgroup analysis, no significant association with PCa risk was found in Caucasians or mixed population. However, L432V polymorphism was significantly associated with increased PCa risk under additive model (OR = 2.38, 95% CI = 1.31–4.33, P = 0.004; Figure S1), recessive model (OR = 2.11, 95% CI = 1.17–3.79, P = 0.01; Figure S2), dominant model (OR = 1.52, 95% CI = 1.14–2.01, P = 0.004; Figure S3), and allelic contrast (OR = 1.52, 95% CI = 1.20–1.92, P = 0.0006; Figure S4) in Asians. For population based studies, there was significant association between L432V polymorphism and PCa risk for additive model comparison (OR = 1.34, 95%CI = 1.14–1.57, P = 0.0003), recessive model comparison (OR = 1.25, 95%CI = 1.09–1.43, P = 0.002), dominant model comparison (OR = 1.25, 95%CI = 1.11–1.41, P = 0.0002), and allelic model comparison (OR = 1.18, 95%CI = 1.09–1.28, P<0.0001). We didn’t find any association in hospital based case-control studies.

#### R48G

There are 6 studies (1647 cases and 1846controls) analyzing the relation between R48G polymorphism and the risk of PCa. In overall population, the Q test of heterogeneity was not significant and we conducted analyses using fixed effect models. Similarly, in subgroup analyses stratified by ethnicity, significant heterogeneity was not detected in Caucasians or mixed population. After stratifying the studies by source of controls, significant between-study heterogeneity only existed in GG+CG versus CC comparison in hospital based case-control studies. We did not detect the association between R48G polymorphism and PCa risk in overall population when examining the contrast of GG versus CC, GG versus CG+CC, GG+CG versus CC, and G versus C. Similarly, no noteworthy association between R48G polymorphism and PCa risk was observed in subgroup analyses by ethnicity or source of controls.

#### N453S

In one study consisting of a Japanese population, no polymorphism on codon 453 was observed as all samples showed wild-type (AA), so 4 independent hospital based studies with a total of 1407 cases and 1499 controls were included in the meta-analysis for N453S polymorphism. The Q-test of heterogeneity was not significant except in allelic contrast. In the stratified analysis by ethnicity, significant between-study heterogeneity was detected in allelic model in Caucasians, but not in mixed population. The data suggested that N453S was associated with PCa risk under dominant model in overall population (OR = 1.18, 95%CI = 1.00–1.38, P = 0.04). For mixed population, there was significant association between N543S polymorphism and PCa susceptibility for dominant model comparison (OR = 1.31, 95%CI = 1.06–1.63, P = 0.01) and allelic model comparison (OR = 1.27, 95%CI = 1.05–1.54, P = 0.01).

#### −13C/T

The association between −13C/T polymorphism and PCa was investigated in 4 independent studies with a total of 1116 cases and 1114 controls. With nonsignificant between-study heterogeneity by Q-test, the analysis was conducted using fixed effect model. Our results do not show any risk of PCa associated with −13C/T polymorphism among subjects of overall population, hospital based studies, or Caucasians.

#### A119S

The meta-analysis for A119S polymorphism was performed based on 4 independent studies (1057 cases and 1018 controls). In the overall analysis, the Q-test of heterogeneity was significant and the random effect model was used except in recessive model comparison. In subgroup analyses, significant between-study heterogeneity only existed in dominant comparison in hospital based case-control studies. The remarkable association with PCa risk was detected in recessive model comparison in overall population (OR = 1.37, 95%CI = 1.04–1.80, P = 0.03). No significant association between A119S polymorphism and PCa risk was found in mixed population or hospital based case-control studies.

### Sensitivity Analysis

Sensitivity analyses were performed to conclude whether modification of the inclusion criteria of the meta-analysis affected the final results. A single study involved in the meta-analysis was deleted each time the analysis was performed to reflect the influence of the individual data set on the pooled ORs. We also assessed the pooled effect of the CYP1B1 polymorphisms on PCa risk within or without the studies that did not follow HWE. Some summary ORs were effectively altered in the sensitivity analysis (Table S1). But most of the corresponding pooled ORs were not materially altered, indicating that our results were statistically robust.

### Publication Bias

Begg’s funnel plot and Egger’s test were performed to assess the publication bias. For R48G, the shape of funnel plots did not reveal any evidence of obvious asymmetry in all comparisons in overall population (Figures S5, S6, S7, and S8), and the Egger’s test was used to provide statistical evidence of funnel plot. The results did not show any evidence of publication bias in all comparisons. For other polymorphisms, similarly, the results did not show any evidence of publication bias in all comparisons. The detailed data were presented in Table 4.

**Table 4:** Egger’s linear regression test to measure the funnel plot asymmetry.

Polymorphism	Additive model		Recessive model		Dominant model		Allelic contrast	
	P value	t	P value	t	P value	t	P value	t
L432V	0.871	−0.17	0.623	0.51	0.925	−0.10	0.974	0.03
R48G	0.661	0.47	0.450	−0.84	0.475	0.79	0.743	0.35
N453S	0.173	2.08	0.158	2.21	0.528	0.76	0.470	0.88
−13C/T	0.245	−1.63	0.463	−0.90	0.549	−0.71	0.481	−0.86
A119S	0.443	0.95	0.347	1.22	0.380	1.12	0.344	1.23

## Discussion

In the recent years, interest in the genetic susceptibility to cancers has led to a growing attention to the study of polymorphisms of genes involved in tumourigenesis. Since the identification of CYP1B1 polymorphisms, growing number of studies suggested that the polymorphisms in the CYP1B1 gene may play an important role in influencing the development of PCa. Epidemiological studies of polymorphisms in CYP1B1 gene, if large and unbiased, can provide insight into the in vivo relationship between the gene and PCa risk. Nevertheless, the results of those studies on the association between−13C/T, R48G, A119S, L432V, and N453S and PCa risk are inconclusive. Some reviewed studies are limited by their sample size and subsequently suffer from too low power to detect effects that may exist. But the pool ORs generated from much larger population can increase the statistical power. In order to provide the comprehensive and reliable conclusion, we performed the present meta-analysis of 14 independent case–control studies, including 6380 cases and 5807 controls.

The present study provides a quantitative analysis of available epidemiologic studies on CYP1B1 −13C/T, R48G, A119S, L432V, and N453S polymorphisms and PCa risk. Our study suggests that −13C/T and R48G polymorphisms may be not risk factors for PCa development. The potential explanation is that the effect of a single polymorphism might have a limited impact on PCa susceptibility. This is in accordance with the hypothesis that PCa is a multi-factorial disease that results from complex interactions between environmental and genetic factors. For N453S, The data suggested that N453S was associated with PCa risk under dominant model in overall population. There was significant association between N543S polymorphism and PCa susceptibility for dominant model comparison and allelic model comparison in mixed population, For A119S, our analysis suggested that A119S was associated with PCa risk under recessive model in overall population. However, in subgroup studies by source of controls and ethnicity, no significant associations were found in any genetic model. For L432V, it was found that the polymorphism was not a risk for PCa on the basis of all eligible studies. In the stratified analysis by source of controls, we found that L432V polymorphism was associated with a trend of increased PCa risk under all genetic models in population based studies. However, significant relation was absent in hospital based studies. When stratifying for the race, no noteworthy associations were observed in Caucasians or mixed population. However, meta-analysis results showed that the L432V polymorphism is significantly associated with PCa susceptibility among Asians for all genetic models. This indicates a possible role of ethnic differences in genetic backgrounds and the environment they lived in. There may be many factors influencing the results, such as differences in populations, selection factors and so on. The complicated nature of PCa allows the same polymorphism to play different roles in PCa susceptibility among different ethnic populations. In the absence of the original data of the reviewed studies, our evaluation of potential interactions of gene-environment with PCa risk was limited. This may explain why previous genetic association studies and some subgroup analyses failed to show an association between these polymorphisms and risk of PCa. Considering the limited studies and population numbers of Africans and Asians included in the meta-analysis, this may increase the risk of false negative findings, any conclusions at overall population level should be interpreted with caution. And above discrepancy we observed may be due to the difference in the source of the controls. We thought the population-based controls were more representative of the general population. Thus, in genetic association studies, the selection of controls and matching status should be carefully considered. If we use the population based controls, we can obtain a higher reliability. Moreover, the above discrepancy might be due to chance because studies with small sample sizes may be underpowered to detect a slight effect or may have generated a fluctuated risk estimate. In the subgroup analyses by ethnicity, incomplete information for mixed ethnicities made it impossible to perform ethnic subgroup analysis of Africans. Thus, additional studies are warranted to evaluate the effect of these polymorphisms on PCa risk in different ethnicities in the future, especially in Africans. Therefore, the results of this study should be interpreted with caution.

Heterogeneity is a potential problem when interpreting the results of the present meta-analysis. Moreover, though there was heterogeneity between the combined studies of CYP1B1 L432V, when we analysed the L432V polymorphism in subgroup analyses, the between-study heterogeneity was effectively removed or decreased. For N453S, there was between-study heterogeneity under allelic model. In subgroup analysis, the heterogeneity was removed in mixed population. For A119S, there was between-study heterogeneity under additive model, dominant model, and allelic model. In subgroup analysis, the heterogeneity was effectively removed or decreased. These results suggest that the heterogeneity may be partly due to source of controls and the variable effects of stratified ethnic subgroups, and some genetic polymorphisms may be associated with risk of some diseases in a specific ethnic subgroup. Another important issue for any meta-analysis is publication bias due to selective publication of reports. In the current study, funnel plot and Egger’s test were performed to evaluate this problem. Both the shape of funnel plots and statistical results did not show publication bias.

Several potential limitations of the present meta-analysis should be taken into consideration. First, although the funnel plot and Egger’s test showed no publication bias and although an exhaustive literature search was done, it is likely that relevant unpublished studies that might meet our inclusion criteria were overlooked. Selection bias for the meta-analysis might have occurred. Second, although all eligible studies were summarized, the total sample size might have not been enough to make a convincing conclusion. When stratified analysis of ethnicity or source of controls was performed, the number of each subgroup was smaller. Thus, the results may be interpreted with care. Third, our result was based on unadjusted estimates due to the absence of available information, while a more precise analysis would be detected if more detailed individual data were available, such as age, smoking status, drinking status and environmental factors. In addition, we did not have detailed individual information on these 5 SNPs, and this made it impossible to make detailed analyses of linkage disequilibrium and the combined effect of various genetic polymorphisms and PCa susceptibility. Further investigations of the haplotypic effect of a gene and the study of multiple polymorphisms in different genes within the same pathway and different pathways are needed.

### Conclusions

In conclusion, although those limitations mentioned previously made the power of this analysis go down, our meta-analysis provides the results based on a number of cases and controls. The results of the present meta-analysis suggest that L432V, N453S, and A119S polymorphisms of CYP1B1 might be risk factors for PCa. Further researches using standardized unbiased methods, and larger numbers of worldwide participants are expected to examine the association to confirm our results, and other possible confounding risk factors like age, life style, and drinking status should also be controlled when it was assessed. Moreover, gene-gene and gene-environment interactions should also be considered.

## Supporting Information

Figure S1 **Forest plot of ORs with 95% CI for CYP1B1 L432V polymorphism and risk of PCa (GG versus CC).**
(PNG)

Figure S2 **Forest plot of ORs with 95% CI for CYP1B1 L432V polymorphism and risk of PCa (GG versus CC+GC).**
(PNG)

Figure S3 **Forest plot of ORs with 95% CI for CYP1B1 L432V polymorphism and risk of PCa (GG+GC versus CC).**
(PNG)

Figure S4 **Forest plot of ORs with 95% CI for CYP1B1 L432V polymorphism and risk of PCa (G versus C).**
(PNG)

Figure S5 **Funnel plots for publication bias for all population in additive model (R48G: GG versus CC).**
(PNG)

Figure S6 **Funnel plots for publication bias for all population in recessive model (R48G: GG versus CC+GC).**
(PNG)

Figure S7 **Funnel plots for publication bias for all population in dominant model (R48G: GG+GC versus CC).**
(PNG)

Figure S8 **Funnel plots for publication bias for all population in allelic model (R48G: G versus C).**
(PNG)

Table S1 **Some corresponding pooled ORs were materially altered in sensitivity analysis.**
(DOC)

Checklist S1 **The PRISMA 2009 checklist.**
(DOC)
