# Supplementary figures and images for: CYP1B1 Polymorphisms and Susceptibility to Prostate Cancer: A Meta-Analysis
Source: PLoS One. 2013 Jul 4;8(7):e68634. doi: 10.1371/journal.pone.0068634 (PMC3701676; doi:10.1371/journal.pone.0068634)

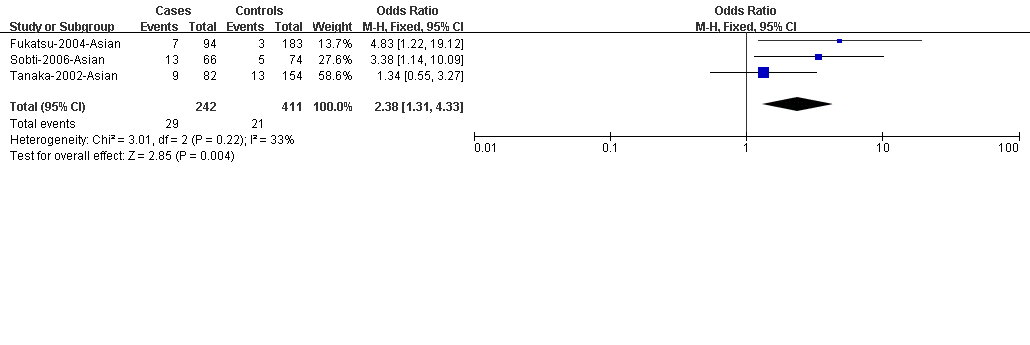

Supplement: Figure S1 — Forest plot of ORs with 95% CI for CYP1B1 L432V polymorphism and risk of PCa (GG versus CC). (PNG) [file pone.0068634.s001.png]

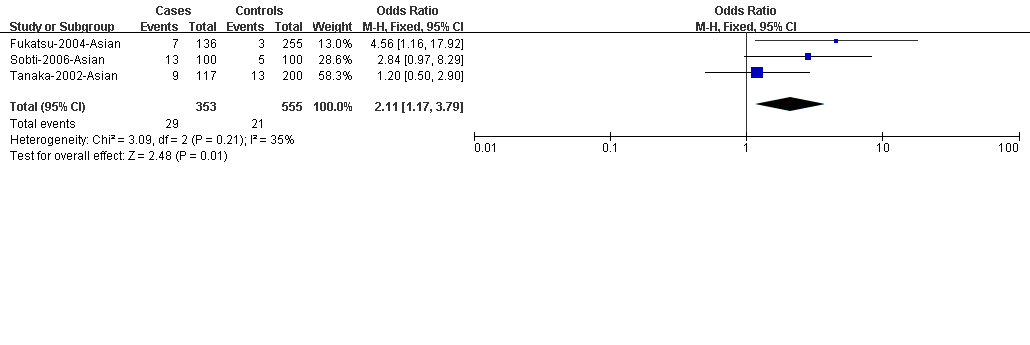

Supplement: Figure S2 — Forest plot of ORs with 95% CI for CYP1B1 L432V polymorphism and risk of PCa (GG versus CC+GC). (PNG) [file pone.0068634.s002.png]

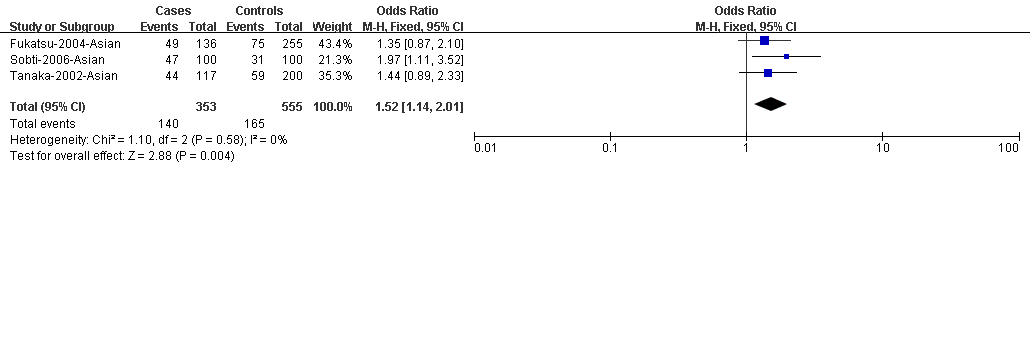

Supplement: Figure S3 — Forest plot of ORs with 95% CI for CYP1B1 L432V polymorphism and risk of PCa (GG+GC versus CC). (PNG) [file pone.0068634.s003.png]

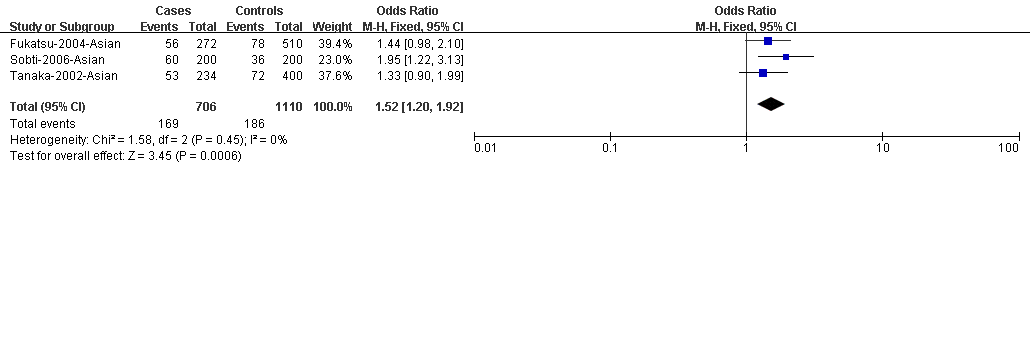

Supplement: Figure S4 — Forest plot of ORs with 95% CI for CYP1B1 L432V polymorphism and risk of PCa (G versus C). (PNG) [file pone.0068634.s004.png]

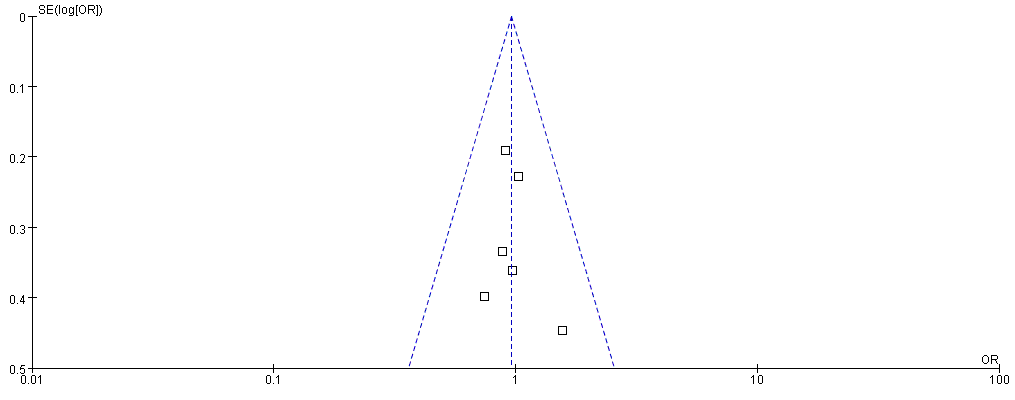

Supplement: Figure S5 — Funnel plots for publication bias for all population in additive model (R48G: GG versus CC). (PNG) [file pone.0068634.s005.png]

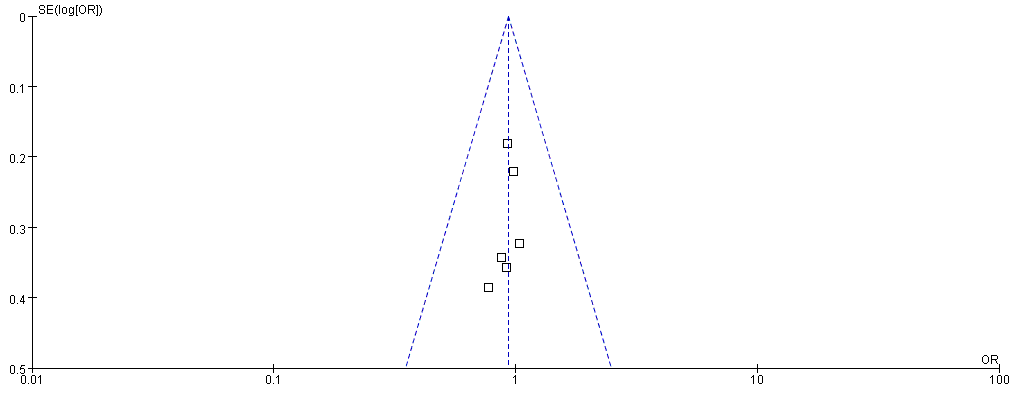

Supplement: Figure S6 — Funnel plots for publication bias for all population in recessive model (R48G: GG versus CC+GC). (PNG) [file pone.0068634.s006.png]

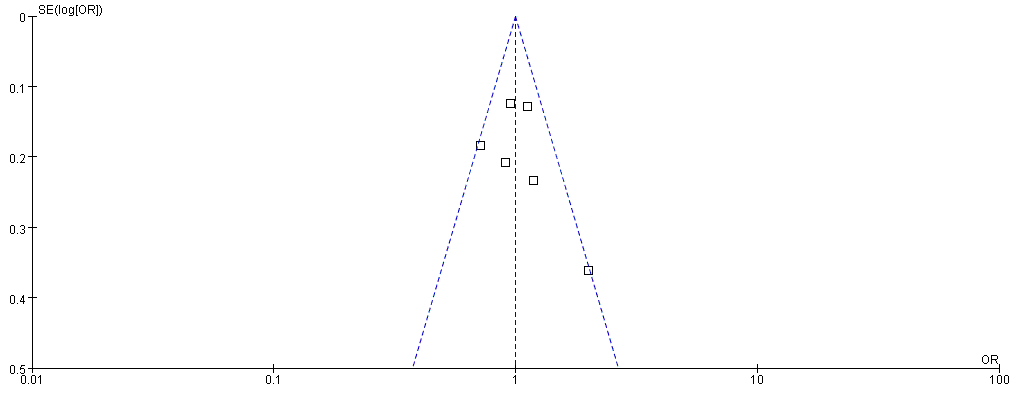

Supplement: Figure S7 — Funnel plots for publication bias for all population in dominant model (R48G: GG+GC versus CC). (PNG) [file pone.0068634.s007.png]

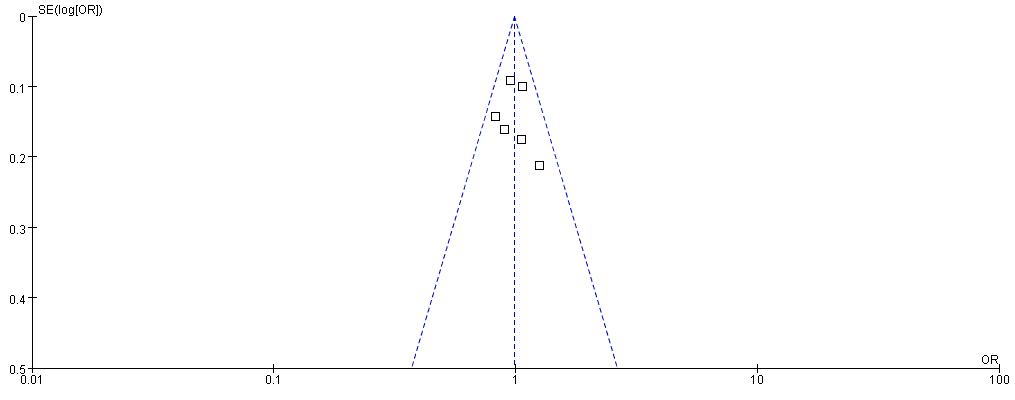

Supplement: Figure S8 — Funnel plots for publication bias for all population in allelic model (R48G: G versus C). (PNG) [file pone.0068634.s008.png]
