# Supplementary material for: CYP1B1 Polymorphisms and Susceptibility to Prostate Cancer: A Meta-Analysis
Source: PLoS One. 2013 Jul 4;8(7):e68634. doi: 10.1371/journal.pone.0068634 (PMC3701676; doi:10.1371/journal.pone.0068634)
Supplement: Table S1 — Some corresponding pooled ORs were materially altered in sensitivity analysis. (DOC) [file pone.0068634.s009.doc]

**Table S1. Some corresponding pooled ORs were materially altered in sensitivity analysis**

|  | OR (95% CI) | P | Ph | OR (95% CI) | P | Ph |
| --- | --- | --- | --- | --- | --- | --- |
| N453S | Dominant model | | | Allelic model | | |
| Overall | 1.18 (1.00-1.38) | 0.04 | 0.37 | 0.97 (0.56-1.68) | 0.92 | <0.00001 |
| Berndt et al. | 1.13(0.92-1.38) | 0.24 | 0.26 |  |  |  |
| Beuten-Hispanic et al. | 1.17(0.99-1.38) | 0.07 | 0.21 |  |  |  |
| Beuten-Non- Hispanic et al. |  |  |  | 1.24(1.05-1.48) | 0.01 | 0.76 |
| Chang et al. | 1.13(0.95-1.34) | 0.16 | 0.38 |  |  |  |
| L432V | Dominant model | | |  |  |  |
| Overall | 1.09 (0.96-1.24) | 0.19 | 0.02 |  |  |  |
| Berndt et al. | 1.15 (1.06-1.25) | 0.001 | 0.16 |  |  |  |
| A119S | Additive model | | | Recessive model | | |
| Overall | 1.59 (0.97-2.61) | 0.07 | 0.05 | 1.37(1.04-1.80) | 0.03 | 0.22 |
| Chang et al. | 1.88 (1.08-3.28) | 0.03 | 0.08 |  |  |  |
| Ciek et al. |  |  |  | 1.36(0.97-1.91) | 0.08 | 0.11 |
| Tanaka et al. |  |  |  | 1.23 (0.92-1.66) | 0.16 | 0.79 |
